# Surveillance for Emerging and Reemerging Pathogens Using Pathogen Agnostic Metagenomic Sequencing in the United States: A Critical Role for Federal Government Agencies

**DOI:** 10.1089/hs.2023.0099

**Published:** 2024-04-16

**Authors:** Diane L. Downie, Preetika Rao, Corinne David-Ferdon, Sean Courtney, Justin S. Lee, Claire Quiner, Pia D. M. MacDonald, Keegan Barnes, Shelby Fisher, Joanne L. Andreadis, Jasmine Chaitram, Matthew R. Mauldin, Reynolds M. Salerno, Jarad Schiffer, Adi V. Gundlapalli

**Affiliations:** Diane L. Downie, PhD, MPH, is Deputy Associate Director for Science, Office of Readiness and Response, US Centers for Disease Control and Prevention, Atlanta, GA.; Preetika Rao, MPH, is a Health Scientist, US Centers for Disease Control and Prevention, Atlanta, GA.; Adi V. Gundlapalli, MD, PhD, is a Senior Advisor, Data Readiness and Response, Office of Public Health Data, Surveillance, and Technology, US Centers for Disease Control and Prevention, Atlanta, GA.; Corinne David-Ferdon, PhD, is Associate Director of Science, Office of Public Health Data, Surveillance, and Technology, US Centers for Disease Control and Prevention, Atlanta, GA.; Sean Courtney, PhD, is a Health Scientist, US Centers for Disease Control and Prevention, Atlanta, GA.; Jasmine Chaitram, MPH, is Branch Chief, US Centers for Disease Control and Prevention, Atlanta, GA.; Reynolds M. Salerno, PhD, is Director, Division of Laboratory Systems, US Centers for Disease Control and Prevention, Atlanta, GA.; Justin S. Lee, DVM, PhD, is a Health Scientist, Division of Global Health Protection, US Centers for Disease Control and Prevention, Atlanta, GA.; Joanne L. Andreadis, PhD, is Associate Director for Science, US Centers for Disease Control and Prevention, Atlanta, GA.; Matthew R. Mauldin, PhD, is Health Scientists US Centers for Disease Control and Prevention, Atlanta, GA.; Jarad Schiffer, MS, is Health Scientists, Office of Readiness and Response, US Centers for Disease Control and Prevention, Atlanta, GA.; Claire Quiner, MPH, MCP, is a Research Public Health Analyst, Social, Statistical, and Environmental Sciences, RTI International, Research Triangle Park, NC.; Pia D. M. MacDonald, PhD, MPH, is a Senior Infectious Disease Epidemiologist, Social, Statistical, and Environmental Sciences, RTI International, Research Triangle Park, NC.; Keegan Barnes is a Public Health Analyst, Social, Statistical, and Environmental Sciences, RTI International, Research Triangle Park, NC.; Shelby Fisher, MPH, is an Epidemiologist, Social, Statistical, and Environmental Sciences, RTI International, Research Triangle Park, NC.

**Keywords:** Metagenomic Sequencing, Pathogen agnostic, Emerging pathogens, Disease Surveillance, Public health preparedness/response, Infectious diseases

## Abstract

The surveillance and identification of emerging, reemerging, and unknown infectious disease pathogens is essential to national public health preparedness and relies on fluidity, coordination, and interconnectivity between public and private pathogen surveillance systems and networks. Developing a national sentinel surveillance network with existing resources and infrastructure could increase efficiency, accelerate the identification of emerging public health threats, and support coordinated intervention strategies that reduce morbidity and mortality. However, implementing and sustaining programs to detect emerging and reemerging pathogens in humans using advanced molecular methods, such as metagenomic sequencing, requires making large investments in testing equipment and developing networks of clinicians, laboratory scientists, and bioinformaticians. In this study, we sought to gain an understanding of how federal government agencies currently support such pathogen agnostic testing of human specimens in the United States. We conducted a landscape analysis of federal agency websites for publicly accessible information on the availability and type of pathogen agnostic testing and details on flow of clinical specimens and data. The website analysis was supplemented by an expert review of results with representatives from the federal agencies. Operating divisions within the US Department of Health and Human Services and the US Department of Veterans Affairs have developed and sustained extensive clinical and research networks to obtain patient specimens and perform metagenomic sequencing. Metagenomic facilities supported by US agencies were not equally geographically distributed across the United States. Although many entities have work dedicated to metagenomics and/or support emerging infectious disease surveillance specimen collection, there was minimal formal collaboration across agencies.

## Introduction

Surveillance and detection of emerging and reemerging pathogens are critical to public health preparedness, outbreak detection, and response. These actions rely on the accurate and timely identification of pathogens in patients, as well as strong, coordinated public–private disease surveillance systems and networks. The COVID-19 pandemic exposed the need for multilevel government coordination to support the identification of and response to current and future biothreats.^1,2^ The *National Biodefense Strategy and Implementation Plan*^3^ provides a path for federal preparedness, including planning, coordination, and sharing of interoperable data between federal entities led by the US Department of Health and Human Services (HHS), and should rely on a strong, integrated federal disease surveillance system, which is currently lacking.^4^

Accurate and timely testing is the foundational basis for identifying new and remerging pathogens, and implementing reliable, unbiased pathogen agnostic diagnostic techniques at scale can provide valuable data.^4^ Advanced molecular techniques, such as metagenomic sequencing, are becoming more prevalent in both the private and public sector. Additionally, because metagenomic sequencing is threat agnostic, it can be performed on specimens from patients with an infectious disease of unknown origin.^5^ Furthermore, metagenomic sequencing may provide data on known and unknown pathogens and can be used to provide complete pathogen genome sequencing, evolutionary phylogenetic analysis, antimicrobial resistance prediction, and information on a pathogen's physical structure.

Pathogen genomic surveillance over the past decade has helped to identify new viral strains; develop diagnostic assays, therapeutics, and vaccines; and monitor and mitigate disease transmission during epidemics and pandemics, notably H1N1 influenza A (2009), Ebola (2014-2016), poliovirus (2014), Zika (2016), and COVID-19 (2019-present).^6-12^ The majority of emerging and remerging human pathogens are zoonotic in origin, necessitating a holistic One Health approach leverages several technologies into a flexible early warning system. This system should be capable of pivoting from a systematic sampling scheme for sustained geographic and demographic representative sampling to a targeted investigation. Such a system would require a data infrastructure capable of integrating molecular results with pertinent metadata and the ability to share, analyze, and report across jurisdictions and sectors. Integration of such complex data into modeling frameworks has the potential to drastically change public health responses in the future.^13,14^ These data can aid in the surveillance of new or reemerging pathogens and improve coordinated, agile, multipronged responses to outbreaks. This system holds especially great promise for responses to future viral threats such as pandemic influenza, respiratory syncytial virus, and zoonotic pathogens.^6,15-18^

Local, regional, tribal, and federal coordination during public health emergencies, such as COVID-19, has at times been challenging. Future networks and data-sharing mechanisms, both vertically and horizontally, could improve information flow and allow for a sound structure to develop.^4,19-21^ Federal, local, tribal, and regional coordination, along with public–private partnerships, would ideally form a web of connectivity, interoperability, and planned disease surveillance that link together as part of a holistic, One Health approach to more quickly identify and respond to future biothreats.^2,22,23^

Although there are important academic and private sector contributions to surveillance and to a broader surveillance network, this study focused specifically on the deployment of metagenomic sequencing techniques among entities supported by the US government. Therefore, to develop a nationwide network, an important first step to determine the current geographic distribution and availability of US government resources, capabilities, and programs.

Assessing resources and infrastructure across federal agencies—including clinical, research, and surveillance programs with metagenomic capabilities, expertise, and knowledge—may provide insight into existing and planned disease surveillance systems to inform the development of more coordinated sentinel surveillance networks. This study aimed to determine which entities (eg, disease surveillance systems, research programs, clinical networks) are contributing to pathogen identification from clinical specimens with suspected unknown infectious etiologies and are part of or supported by the US government. Furthermore, the aim was to determine which of these entities use metagenomics in their approach and the origins of the specimens being tested.

## Methods

### Entity Landscape Analysis

We began by conducting a landscape analysis of federal agency websites for publicly accessible information on the availability and type of clinical, research, and surveillance programs, with a focus on pathogen agnostic testing and details on the flow of clinical specimens and data. The identification of entities, institutions, programs, and networks with involvement in pathogen agnostic testing relied primarily on the research team's institutional knowledge of disease or pathogen surveillance and research efforts across US government agencies. For the purposes of the landscape analysis, metagenomics was defined as a pathogen agnostic, unbiased, sequencing technique that produces sequences of all microorganisms found in each specimen.

An entity was defined as an agency, funding program, clinical network, division, center, research network, public–private partnership, or program that is part of or supported by US government agencies. This definition does not include public health laboratories that service states and counties in the United States and may receive federal funding. Entities varied widely in size and scope and ranged from small laboratories within universities to large disease surveillance networks with hundreds of partners. The level of granularity chosen to use when defining an entity depended on the information publicly available, the number of entities with unique contributions within a network, and the relevancy of the entities' activities to the review. This search did not include any entities outside of the continental United States.

The initial set of entities was identified using information sourced from key informants, networks, and literature reviews. We used the following 3 inclusion criteria: (1) entities that are part of, funded by, and/or in direct coordination with a US government agency; (2) entities that perform metagenomics, per our definition, for pathogen detection/characterization; and (3) entities that collect or have access to clinical specimens from the United States, and that have potential utility for a sentinel surveillance system for emerging infectious diseases, such as systemic specimen collections or high-priority specimens for emerging infectious diseases (eg, from immunocompromised patients, international travelers). For entities that perform metagenomics, exclusion criteria were any metagenomic facilities located outside of the United States. For entities that collect relevant clinical specimens, exclusion criteria were: (1) entities with only international specimen collection or (2) entities that have access only to data, not physical specimens.

The descriptions of each entity's mission and activities were collected from their websites. For entities that performed metagenomics, the information collected included the instrumentation used (eg, Illumina MiSeq), sample sources, location of instrumentation, how metagenomic data are utilized, and funding sources. For clinical specimen flow, the specimen type, origin population, and geography of the sampling catchment area were collected. When these details were not provided on an entity's website, we performed a scan of peer-reviewed publications. For each entity, information was extracted from up to 3 publications. These data collections occurred in the first quarter of 2023.

### Expert Interviews

The landscape analysis was supplemented by a review of results by expert representatives from the federal agencies who provided entity details and filled in knowledge gaps, including specimen sources, communication networks, and origin of clinical samples in pathogen agnostic testing workflows. Semistructured questions aimed to elicit feedback on other aspects and agencies in the landscape of metagenomic testing, resources required for a potential national sentinel surveillance system of emerging infectious diseases, and opinions on the challenges of designing and operationalizing a coordinated network. In the process of collecting this information from both the publicly available resources and representatives from the federal agencies, additional relevant entities were identified.

## Results

**Figure 1.**
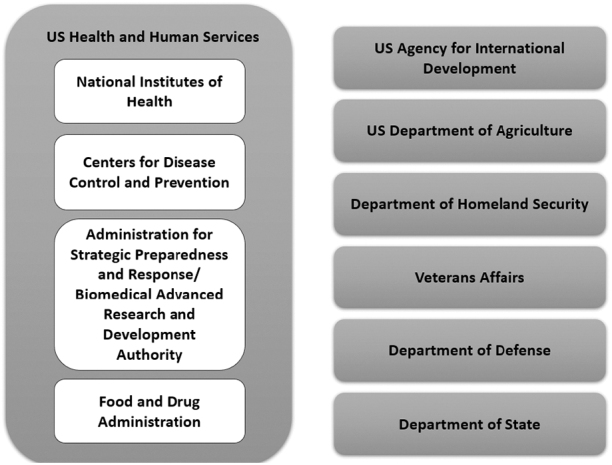
Entities described in the landscape analysis are part of or directly supported by these US government agencies.

In total, 146 entities across 10 US government agencies were reviewed for potential contributions to a sentinel surveillance system for emerging infectious diseases (Figure 1). The agencies involved in these activities were: HHS (including the National Institutes of Health, Centers for Disease Control and Prevention, Administration for Strategic Preparedness and Response, Biomedical Advanced Research and Development Authority, and the Food and Drug Administration), US Agency for International Development, Department of Homeland Security, Department of Veterans Affairs, Department of Defense, Department of Agriculture, and the Department of State. In general, the entities included clinical networks, research networks, disease surveillance programs, centers, and divisions.

### Geographic Scope of Facilities

**Figure 2.**
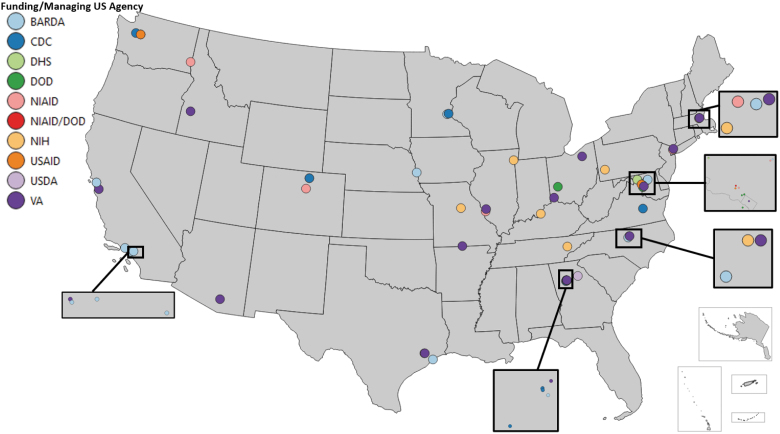
Map of entities with metagenomic capabilities based on a review of publicly available information on entity websites and publications. Abbreviations: BARDA, Biomedical Advanced Research and Development Authority; CDC, Centers for Disease Control and Prevention; DHS, Department of Homeland Security; DOD, Department of Defense; NIAID, National Institute of Allergy and Infectious Diseases; NIH, National Institutes of Health; USAID, US Agency for International Development; USDA, US Department of Agriculture; VA, Department of Veterans Affairs.

Given the high cost and specialized nature of metagenomic assays, this study sought to understand the geographic distribution of entities with this capability, across the United States. Geographic distribution is observably nonuniform (Figure 2). On the West Coast, a high density of entities was found in southern and northern California; Seattle, Washington; and in the state of Idaho. There is a paucity throughout other western states (eg, Kansas, New Mexico, North Dakota, South Dakota, Wyoming). The midwestern states (eg, Illinois, Minnesota, Missouri) and many Eastern Seaboard states (eg, Georgia, New York, North Carolina) have some entities, in low densities. The number and density of these entities is highest on the East Coast (eg, Georgia, New York, Washington, DC). Based on our review of publicly available information, more than half of the 50 states do not have metagenomic capabilities funded directly or indirectly by the US government.

### Metagenomic Activities and Platforms

Contributions of the entities were categorized into 2 groups of activities: (1) performs metagenomic sequencing, and (2) potentially collects specimens via a systematic means or has access to specimens of high interest that are sent to other entities for pathogen sequencing (eg, specimens from immunocompromised populations and travelers) (Table 1). Of the 146 federal entities reviewed, 58% (n=84) performed metagenomics, 51% (n=74) collected specimens, and 21% (n=30) performed metagenomics and collected specimens. Specimen types were noted to be primarily blood products and sputum, as well as respiratory specimens (such as bronchoalveolar lavage), saliva, urine, stool, cerebrospinal fluid, synovial fluid, bone, lymphatic tissue, and tissue from skin lesions. In our review of the entities, we abstracted approximately 600 data points—including mission, metagenomic capabilities, metagenomic techniques, instrumentation, manufacturers, geographic region, and specimen collection—from 140 external resources, including websites and scientific publications.

**Table 1.** Metagenomic Activities of Entities Identified During Landscape Analysis

Potential Contribution	No. of Entities
Performs metagenomics	84
Potentially collects relevant specimens	74
Performs metagenomics and collects relevant specimens^a^	30
Does not perform metagenomics or collect relevant specimens	18

^a^ The 30 entities that perform metagenomics and collect relevant specimens are included in the number of entities in the previous rows.

The use of sequencing instrumentation was captured where reported, with many entities indicating multiple platform types. While not mutually exclusive, Illumina products accounted for 81% of reported platforms (Table 2). Although many entities have work dedicated to metagenomics and/or support emerging infectious disease surveillance specimen collection, there was minimal formal collaboration across agencies. Additionally, there were no formal efforts to coordinate and collate results from these networks to provide surveillance reports for emerging and reemerging pathogens and biothreats at the regional or national level.

**Table 2.** Sequencing Platforms Used for Metagenomic Analyses

Manufacturer	Platform	No. of Entities Reporting Use
Illumina	MiSeq	25
	NextSeq	15
	HiSeq	8
	NovaSeq	8
	SmartSeq	1
	MiniSeq	1
Oxford	Nanopore	5
	NanoString	2
Bionano	Saphyr	1
Pac Bio	Pac Bio Sequel	2
	BugSeq	1
NA	Other	3
	Not Specified	51

Notes: Of the entities identified that reported performing metagenomics, these sequencing platforms were reported to have been used or available. Sequencing platforms used were not mutually exclusive. Information was sourced from entity websites or from their peer-reviewed publications, for entities that used metagenomics. Abbreviation: NA, not available.

We identified 9 funding sources (Table 3) for metagenomic facilities that supported multiple entities, based on information provided in the acknowledgment and funding sections of representative peer-reviewed publications. An additional 22 domestic and 7 international funding sources were acknowledged in single studies or publications.

**Table 3.** Funding Sources Acknowledged in the Landscape Analysis

Funding Source	Frequency
NIH	9
CDC	4
Active Bacterial Core (CDC)	3
NIAID	3
The Broad Institute	2
Howard Hughes Medical Institute	2
Abbott Viral Discovery Award	2
AFHSC-GEIS	2
William K. Bowes, Jr. Foundation	2

Note: These funding sources were extracted from publications from entities included in the landscape analysis that used metagenomics. Abbreviations: AFHSC-GEIS, Armed Forces Health Surveillance Center, Division of Global Emerging Infections Surveillance and Response System Operations; CDC, Centers for Disease Control and Prevention; NIH, National Institutes of Health; NIAID; National Institutes of Allergy and Infectious Diseases.

## Discussion

Results indicate significant potential resources for emerging infectious disease surveillance, with numerous federal agencies and programs currently using or funding metagenomic sequencing and related techniques to identify emerging and reemerging pathogens. Despite the existence of clinical and research networks funded or operated by federal agencies, there is currently no nationwide sentinel surveillance system that leverages pathogen agnostic metagenomic sequencing for identification of emerging, reemerging, and unknown infectious disease pathogens. There are variations in organizational structure and reporting detail between entities, and although individual entities were assessed, documenting geographic location and sequencing technology was difficult in some instances, as many entities did not report this information or aggregate it at higher levels.

Findings from this study revealed several reasons why it would be challenging to successfully implement a nationwide pathogen surveillance system based on metagenomic sequencing. First, physicians may not know where to send specimens for pathogen agnostic metagenomic sequencing when conventional tests are inconclusive and there remains a high suspicion of an infectious etiology. At present, the options appear to be limited and further understanding of metagenomic sequencing results and coordination in clinical, research, and pathogen surveillance activities is needed. For example, metagenomic sequencing may be possible only if there is an existing research protocol at the hospital where the patient is admitted, or if a physician knows of an academic researcher or program/person to contact. Second, more information is needed regarding which laboratories are certified by Clinical Laboratory Improvement Amendments (CLIA) and which metagenomic sequencing tests are validated for individual patient diagnostic use. CLIA certification is not required for disease surveillance purposes, but many of the systems identified in this study were directed at individual patient-level diagnostics rather than population surveys. Third, there are not many laboratories (eg, public health, clinical, commercial, academic) that routinely offer pathogen agnostic metagenomic sequencing for clinical diagnostics, because payment models may not be well-defined, instruments are expensive, and data analytic requirements are advanced.

Many areas of the US federal government were identified as having metagenomic sequencing capabilities and emerging and reemerging infectious diseases programs, laboratories, and/or initiatives. Based on our review, overt coordination of these efforts was not apparent. Coordination at the federal level is an essential first step to improve national detection, data sharing, and reporting of emerging and reemerging pathogens, pandemics, and biothreats via advanced molecular testing techniques. Operationalizing such a system would likely require investments in instrumentation, specimen and data-sharing networks, and training/education to strengthen capacity among clinicians, laboratory scientists, and bioinformaticians. Current regulations and data-sharing practices need to be strengthened to ensure that pathogen surveillance data are not identifiable at the individual level and do not compromise personal privacy.^24,25^ National strategies that advocate for timely detection of current and emerging infectious diseases are a critical input for improved situational awareness and decisionmaking at many levels (eg, state, local, tribal) and with private sector partners.^26^

Supporting pathogen genomic analysis across public health settings would be well served by the creation of a unified bioinformatics ecosystem to increase the reproducibility, accessibility, and auditability of data.^2^ Increased capacity for using metagenomics for pathogen discovery is likely; therefore, it will be necessary to have new assays, platforms, funding programs, and methods for standardization to ensure clear documentation and communication among participating entities. Specifically, pathogen genomic sequencing and analytical data-sharing capacities, policies, and capabilities are necessary preparedness elements to mitigate nationally and/or internationally significant biological threats.^3^

As knowledge of advanced molecular techniques and technology evolves over time, an agile, flexible network that continues growth and investigation in these areas will be necessary. While these techniques show significant promise, they are not widely available beyond federal entities, certain academic centers, and large health systems because of the inherent cost, equipment, and capacity-related challenges. Enabling more widespread implementation and further examination of local/regional networks to enhance efficiencies are critical. Consistent resources are needed to create and maintain robust sentinel disease surveillance systems, including well-funded and equipped laboratories, trained clinicians, clear protocols, bioinformatics, improved reference databases, and data management systems.

A potential limitation of this study is that some federal agencies may have been disinclined or unable to share current or future funded activities, which would result in an incomplete picture of institutional use. To mitigate this limitation, results were correlated with other sources, including agency partners and expert interviews, to provide a more comprehensive look at the use and capabilities of advanced molecular testing across federal agencies. Additionally, this study relied on publicly available information at a particular point in time, and we recognize this field is dynamic and changing. In addition to entities with little publicly available information, there may be entities that have no public presence or otherwise were not identified in our search. A landscape report can only capture a snapshot in time, and this landscape is constantly changing as entities develop metagenomic capabilities.

There are 2 reasons to suspect a current period of rapid change. First, as technology becomes more refined, usage expands, and economies of scale emerge, there will likely be more entities that perform metagenomics for pathogen discovery, new assays, platforms, funding programs, and networks. Second, the COVID-19 pandemic has increased both the demand for genomic sequencing and the supply of federal funds for that purpose. The pathogen agnostic sequencing landscape is in considerable flux. Although it may be a limitation of this study, it bodes well for the future of preparedness and readiness efforts.

Further research from the perspective of clinical care, public health, commercial laboratories, and academic researchers may further elucidate the landscape and help shape a strategy and operational plan to achieve a national sentinel surveillance system for emerging, reemerging, and unknown infectious disease pathogens. There are several existing federal entities and programs that may be leveraged to assemble a systematic and representative sentinel surveillance system.

## Conclusion

This article is a first step in improving the understanding of the current breadth and scope of pathogen agnostic advanced molecular testing capabilities funded and/or operated by federal government agencies. Identification and coordination of US government capabilities lays the groundwork for future response planning and further research and strategies in this field. Although challenges remain in implementing advanced molecular techniques at scale, results from these techniques are critical to sentinel surveillance monitoring and outbreak preparedness. Development of sustained support and policies and standards for strengthening sentinel surveillance networks will likely improve coordination of reporting between entities and aid in the identification and detection of emerging and reemerging pathogens at a national level.
